# Catalyzing social change: Does concentration encourage action?

**DOI:** 10.1371/journal.pone.0277934

**Published:** 2022-12-28

**Authors:** Jonah Berger, Joshua Conrad Jackson, Ceren Kolsarici

**Affiliations:** 1 Marketing Department, The Wharton School, The University of Pennsylvania, Philadelphia, PA, United States of America; 2 Management Department, Kellogg School of Management, Northwestern University, Evanston, IL, United States of America; 3 Marketing Department, Smith School of Business, Queen’s University, Kingston, Ontario, CA; Universidade Estadual de Maringa, BRAZIL

## Abstract

Countless social problems demand solutions, from climate change and gun control to poverty and systemic racism. But while some of these problems inspire action (e.g., “Black Lives Matter” and “Me Too” movements), most fail to gain traction or inspire new policy. Why do some problems garner more attention and response? We suggest that the relative timing of related events may play an important role. Specifically, action may be more likely when related events are concentrated in time. A multi-method investigation tests this possibility. Study 1 borrows a modeling strategy from the economics and marketing literatures to examine a particularly important domain: gun control. Analysis of over 40 years of gun control legislation finds that, even after controlling for the frequency of mass shootings, bills are more likely to be proposed (and passed) when shootings are concentrated in time. Study 2 further tests concentration’s causal impact and demonstrates that concentration increases support against sexual assault. These findings illustrate how a modeling approach commonly used to study advertising goodwill can be applied to a broader set of situations, suggest why some social problems are more likely to catalyze action, and shed light on drivers of social movements and collective action.

## Introduction

Consumers are surrounded by countless social problems that demand solutions, from climate change and gun control to poverty and systemic racism. But while some of these problems inspire action (e.g., the “Black Lives Matter” and “Me Too” movements), most fail to gain traction or inspire new policy. These trends are important to understand from a social justice standpoint, but they also raise puzzling questions about cultural change itself. Why do some problems garner attention and generate response while others do not? And what leads consumers and institutions to address problems that have long gone unnoticed?

Across the social sciences, researchers have long been interested in cultural change, social movements, and collective action [1–8]. Much of this work has focused on who delivers messages [9, 10], how these messages are framed [11, 12], and whether movements are seen as widely supported [2, 13].

But while research has focused on *who* communicates, or *how* something is framed, there has been less attention to whether responses are shaped by *when* related events occur. More frequently occurring problems, for example, likely encourage action [14]. Lawmakers may be more likely to propose environmental regulation, for example, if there are three oil spills in a year rather than just one.

Beyond the frequency of related events, however, we suggest that their relative *timing* may also play a crucial role. Three oil spills may occur in a year, but they could be concentrated in time (i.e., right after one another) or more spread out (i.e., over months). Which would be more likely to drive action?

Some work suggests that temporal separation may be more likely to encourage change. Persuasion research suggests that concentrated exposure can have diminishing marginal returns [15, 16] and advertisements are often spaced out over time to prevent “wearout” and encourage purchase [17, 18]. Similarly, concentration may lead to inferences that a social issue is localized in time; it was a problem in the past but not anymore.

Anecdotal evidence, however, hints at the possibility that concentration might actually encourage action. Sexual harassment has been an issue for decades, and the phrase “Me Too” originated in 2006, yet the movement didn’t take off until late 2017 when multiple women shared sexual harassment stories following allegations against Harvey Weinstein. Landmark legislation has also tended to follow closely timed events. A series of high-profile protests, for example, and stories of police brutality in response to these protests partly encouraged the 1964 Civil Rights Act [19].

This anecdotal evidence suggests an intriguing “concentration hypothesis:” even holding frequency constant, action may be more likely when related events are temporally concentrated. Three high-profile instances of sexual harassment may be more likely to catalyze response, for example, if they occur soon after one another. Indeed, while this hypothesis may seem to contradict the fact that repeated ad viewing leads to wearout [17], wearout reverses when consumers view *different* advertisements for the same product, which may better approximate how consumers perceive problematic events [18]. Overall then, we suggest that concentrated events may be more likely to encourage action.

A multi-method investigation tests the concentration hypothesis in two important domains: Gun control and sexual assault. In Study 1, we borrow an approach used in the economics and marketing literatures to estimate consumer responses to advertising [20–22]. While such carryover models have not been previously applied to cultural change, they are particularly useful in this context because they 1) allow repeated exposure over time to be modeled as a form of stock buildup, and 2) do this dynamically accounting for forgetting due to passing time. Instead of advertising goodwill accumulation, we use this approach to model *attention* to mass shootings. To further test concentration’s causal impact, Study 2 uses an experiment. We manipulate the concentration of news articles about sexual assault and test how it impacts support for the issue.

## Empirical analysis of concentration in mass shootings

Every year, tens of thousands of people in the United States die from firearm-related deaths. Mass shootings, or cases where four or more people die [23], have also become more prevalent. Nevertheless, gun control remains a controversial issue, and Congress has vacillated between introducing and rolling back gun legislation.

To test whether concentration encourages action—operationalized here through institutional action—we estimate the relationship between mass shootings and gun control legislation in America from 1980 to 2020. Over this 480-month period, there were 230 mass shootings, over 1500 fatalities, and over 700 gun-control bills proposed. Multiple mass shootings should translate to a higher likelihood of legislation being proposed and passed into law, but if the concentration hypothesis is correct, there should be more legislation proposed (and passed) when shootings are concentrated in time.

Testing the concentration hypothesis requires not only examining whether shootings have an immediate impact on bills, but also whether shooting incidences in the past encourage bills in the future and whether the time between shootings shapes any such carryover. By borrowing carryover models from economics and marketing [20–22], we are able to model this temporal interdependence.

Note that, while one recent paper [14] examined the role of the frequency of mass shootings on firearm bills proposed, it did not investigate concentration. Consequently, it remains unclear whether or how the *timing* of related events, after controlling for their frequency, might shape action. Further, by conceptualizing *attention* as a dynamic construct that accumulates over time affected by the history of shooting events, rather than being linked to a single shooting incidence, we examine how it can build and dissipate over time.

### Shootings data

The FBI defines mass shootings as events which have at least four firearm fatalities, excluding the offender(s), and which do not take place in the process of some unlawful act such as burglary or organized crime [24, 25]. This definition is important because one- or two-person shootings are typically considered domestic disputes and do not receive media attention. Further, shootings involving organized crime or burglary often receive media attention for reasons other than possession of firearms.

To identify shootings, we combined data from various existing lists (e.g., Mother Jones and The New York Times) with manual searches. For manual searches, we used the different newspaper archives (e.g., the New York Times and Washington Post), searched for keywords (i.e., “shooting,” “mass shooting,” and “gun violence”) and examined any results to see if they described a mass shooting that met the FBI’s definition. We focus on shootings between January 1980 and December 2019, as before that time period, there was less consistent documentation. In total, there were 230 mass shootings.

For each shooting, we recorded the date, number of fatalities, and shooter age. For shootings with involved multiple shooters, each shooter’s characteristics were recorded. Concentration was measured by the distance (i.e., days between) shootings, reverse scored so that lower values indicated greater concentration. Note that the shootings data is in the form of an irregular time series and the time between shootings vary significantly. In fact, this variation gives rise to our core research question, examining the role of proximity of shootings over time (i.e. concentration) on legislative action. See S1 Table for descriptive statistics.

### Gun control legislation data

Information on gun control legislation was collected from govtrack.us. We searched for all bills containing the words “gun” that were proposed since the 1979–1980 congress. Reading the title and text of the bill clarified whether it was at least partially aimed at gun control, and ambiguous cases were resolved through discussion between co-authors. Bills that did not focus on gun control in the US were excluded (e.g., bills honoring victims of gun violence or aimed at reducing gun violence outside of the US). In total, there were 713 separate pieces of legislation.

Bills were coded as passed (1) or not passed (0) based on the “latest action” variable that govtrack provides. The 2013 amendment of the Safe Schools Act of 2013, for example, was passed into law and improved the availability of records to the National Instant Criminal Background Check System and initiated new regulation on the trafficking of illegal firearms.

### Modeling framework

Building on Nerlove and Arrow (1962) advertising goodwill accumulation model, we first model attention to mass shootings as a function of key shooting characteristics such as frequency and the number of fatalities. Next, we model firearm related bills (proposed and passed) as a function of attention, concentration between mass shootings, and a series of control variables (e.g., time since last bill, shooter age and number of shooters).

It is worth highlighting a couple of points here. First, it would be quite challenging, if not impossible, to come up with an error free measure of the comprehensive attention mass shootings receive from the media, legislature, and public at large. Consequently, rather than imprecisely measuring attention to shootings, we model it as an unobserved construct (i.e. latent). This allows us to formulate attention theoretically based on forgetting behavior and message wear-in and wear-out, as established in the economics, psychology and marketing literature (see [20, 26, 27]). Second, legislative action is unlikely to take place on the same day (or even week) as a particular shooting, which puts emphasis on the importance of the aggregation window in the analysis. We use monthly aggregation since smaller data frequencies (i.e., daily or weekly) do not provide enough variance in the number of shootings (i.e., the coefficient of variation for the number of shootings is 0 and 0.18 for daily and weekly aggregation windows respectively, compared to 0.56 for monthly) which causes identification problems. The relationship between concentration and bills, however, remains the same using broader windows of aggregation (e.g., bi-monthly or triweekly) as we discuss under robustness checks.

Mathematically, we express the attention construct, modeled as a form of stock buildup as shown in Eq (1):

$$\Delta A_{t}=\alpha {f(X}_{t})-\delta A_{t-1}+\varepsilon_{t},$$ (1)

where *A*_*t*_ is attention to mass shootings at month *t*, Δ*A*_*t*_ denotes the change in attention between *t* and (*t*−1), *δ* is the decay or forgetting rate, *X*_*t*_ is the vector of covariates such as number of shootings and total number of fatalities in month *t*, *f*(.) denotes the functional transformation for these focal variables, and *α* is the vector of coefficients measuring their effect on the attention to mass shootings. *ε*_*t*_ is the error term capturing the effect of factors not explicitly included in the model and assumed to be *i*.*i*.*d*. following *N*(0, *Q*).

Eq (1) implies that the attention growth rate, Δ*A*_*t*_, changes as a function of number of shootings and fatalities and decreases due to forgetting, which is proportional to the level of attention that is already built up at *t*−1. As one can see from Eq (1), in the absence of mass shooting events at a particular time period, the attention stock will merely decay (at the rate −*δA*_*t*−1_) as no build up takes place (i.e. *αf*(*X*_*t*_) = 0). We rewrite Eq (1) as the following:

$$A_{t}=\alpha f(X_{t})+(1-\delta )A_{t-1}+\varepsilon_{t},$$ (2)

During periods where no shooting takes place, attention decreases until the next incidence, which adds to the attention stock proportional to the vector of covariates, *X*_*t*_. Given the attention formulation in Eq (2), we model the monthly number of proposed bills as:

$$B_{t}=A_{t}+\beta Z_{t}+v_{t},$$ (3)

Where, *B*_*t*_ is the proposed number of bills in month *t*, *A*_*t*_ is the attention to mass shootings, *Z*_*t*_ is the vector of covariates including our focal variable of concentration, operationalized by average days between shootings, as well as control variables such as time since last bill, average shooter age, average number of shooters and a time trend. *β* is the vector of coefficients measuring the impact of these covariates. *v*_*t*_~*N*(0, *R*) is the *i*.*i*.*d*. error term that captures the effects of factors not included in the model.

As mentioned earlier, we allow for the focal shooting variables (i.e. frequency and number of fatalities) to potentially impact attention in a nonlinear form, denoted by *f*(.) in Eqs 1 and 2. To this end, we estimated 9 different model specifications allowing for all combinations of linear, convex, or concave transformations of the shooting frequency and number of fatalities variables. A concave specification of shootings and convex specification of total fatalities provided the best fit (i.e., highest Log Likelihood, lowest AIC, and BIC, S2 Table), which we use in our proposed model, but the results are robust across specifications.

Eq (4) presents this model where number of shootings (i.e. *X*_1*t*_) and fatalities (i.e. *X*_2*t*_) enter the model in concave and convex forms, respectively.


$$A_{t}=\alpha_{1}\surd X_{1t}+\alpha_{2}X_{2t}^{2}+(1-\delta )A_{t-1}+\varepsilon_{t},$$ (4)

Due to the fact that traditional regression approaches lead to biased estimates in handling time series data [21] and the latent nature of the attention variable, we use a state-space approach and the Kalman Filter [22, 28, 29], which allows us to simultaneously estimate the *unobserved* attention to mass shootings (*A*_*t*_) and how it relates to bills proposed. Eqs (3) and (4) constitute the measurement and process equations of the filter, respectively. For detailed estimation steps and Kalman Filter iterations, the interested reader can refer to Harvey (1990).

Note, we rely on this modeling approach because it is designed to examine the impact of concentration, but we don’t mean to suggest that advertising effects on purchase and mass shooting effects on bills are driven by the exact same mechanisms. Indeed, advertising exposures could affect purchase through conveying additional information, keeping something top of mind, or signaling quality. While some of these mechanisms (e.g., quality signaling) are obviously irrelevant in the gun legislation domain, others (e.g., top of mind awareness) may play a role. Regardless of whether the mechanisms are identical, though, the modeling structure is still useful, and we further discuss potential mechanisms in the General Discussion.

### Results

**Fig 1:**
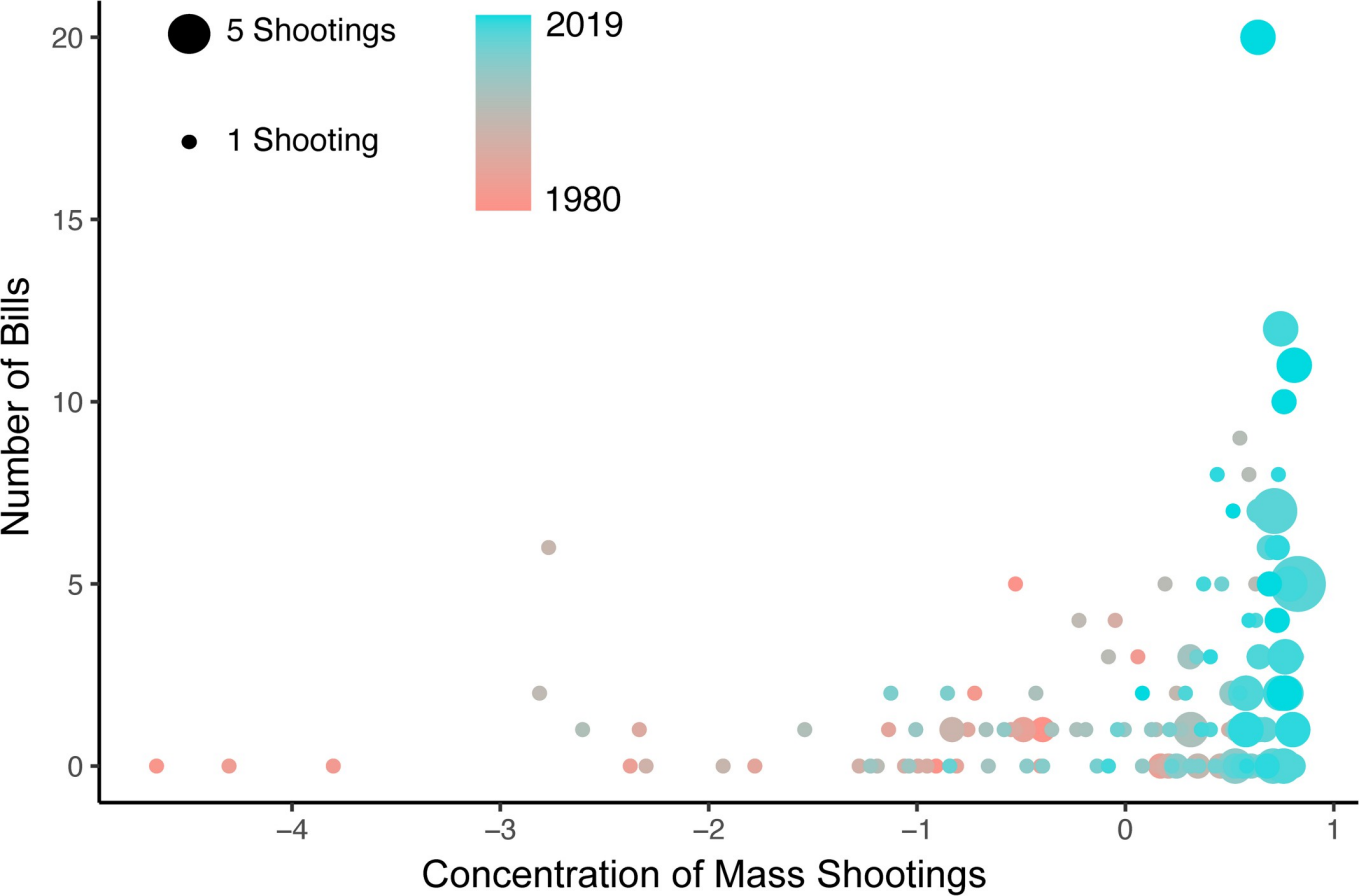
How gun control bills relate to frequency and concentration of mass shootings*. *Node size is number of shootings in a month. Node color is year. Concentration is a standardized index where higher values indicate fewer average days between shootings.

How does the concentration of mass shootings relate to the introduction of gun control legislation? Fig 1 plots gun control bills as a function of concentration, number of shootings, and time. This model-free evidence suggests a positive relationship between concentration and number of bills, but also provides evidence for the importance of controlling for the focal shooting related variables such as the number of shootings.

Table 1 displays the parameter estimates from the Kalman Filter. Starting with the simplest model (Table 1, Model 1), in addition to an effect of frequency (i.e., there are more bills in months with more shootings), results support the concentration hypothesis. When shootings are more concentrated (i.e. fewer days between them), more bills are proposed. Increasing concentration of mass shootings (i.e., reducing the days between shootings) by one standard deviation, for example, increases the proposed number of bills by 25.4%.

**Table 1:** Concentration of mass shootings and gun control bills proposed.

	Model 1	Model 2	Model 3
Concentration	.004***	.004*	.004*
Number of Shootings	.55**	.71*	.94*
Carryover Effect	0.79***	.66***	.66***
Number of Fatalities		.65*	.65*
Time Since Last Bill		.39***	.40***
Average Shooter Age		-.02*	-.02*
Number of Shooters		.09	.09
Time Trend			.00
Log Likelihood	-637	-614	-614
AIC	1284	**1246**	1248
BIC	1305	**1284**	1290

*p < .05

**p < .01

***p < .001

Results persist including a variety of controls. Maybe concentrated shootings have more fatalities, for example, or younger perpetrators, both of which might encourage bills. The results persist including all these aspects (Table 1, Model 2). While more fatalities, younger shooters, and more time since the last bill are all associated with bills (e.g., a standard deviation increase in fatalities is associated with a 3.26% increase bills), even controlling for these and other factors, bill are more likely when shootings are more concentrated.

Alternatively, one could be concerned that time was driving the results. Maybe there is a recent rise in the concentration of shootings, for example, as well as bills, and this is driving the perceived relationship. Casting doubt on that possibility, however, the results also persist controlling for a time trend (Table 1, Model 3). Results are also robust to different levels of aggregating the data (S3 Table).

We also investigated reverse-causality. Rather than mass shootings driving the number of bills proposed, one could argue that the causal arrow somehow goes in the opposite direction. Maybe bill proposals somehow cause mass shootings by unintentionally antagonizing potential shooters. Ancillary analyses, however, cast doubt on this possibility. Treating the number of bills and time since last bill as independent variables and number of shootings as the dependent variable finds that both predictors are insignificant (ps > .30). Similar results persist when using shooting concentration as the dependent variable (ps > .30). Consequently, there is no evidence for reverse-causality.

While the results so far are consistent with the theorizing, one could wonder whether they simply reflect interest, rather than action. Maybe concentration encourages bills to be proposed but doesn’t actually result in change. If consumers have deeply divided views, for example, or see downsides to gun control this may lead to a deadlock in attempts to address the problem. Indeed, which party is in power shapes the type of legislation that gets passed [14].

Consequently, to provide a stronger test of concentration’s impact, we examined whether bills are actually passed into law. Results demonstrate that the effect of concentration extends into action (Table 2). When shootings are more concentrated, bills are more likely to be passed. In fact, 1 standard deviation increase in shooting concentration leads to a 33.9% increase on number of bills passed, suggesting a larger impact than that on bills proposed. Thus, while political divisions certainly shape policy, in this case, concentration is enough to not only drive interest but, more importantly, impact actual change.

**Table 2:** Concentration of mass shootings and gun control bills passed into law.

	Model 1	Model 2	Model 3
Concentration	.001**	.001*	.001*
Number of Shootings	.03	.05	.00
Carryover Effect	0.63***	.53***	.65***
Number of Fatalities		.00	.00
Time Since Last Bill		.01	.01
Average Shooter Age		-.00	-.00
Number of Shooters		-.01	.00
Time Trend			.00
Log Likelihood	-186	-186	-186
AIC	382	390	483
BIC	403	428	434

*p < .05

**p < .01

***p < .001

### Discussion

Analysis of 40 years of mass shootings and gun control legislation suggests that concentration encourages action. Even including a variety of controls (i.e., shooting frequency, fatalities, and time), gun control legislation is more likely to be proposed and passed when mass shootings are concentrated in time. A standard deviation increase in the concentration of shootings within a month leads to a 25.4% increase in the number of bills proposed.

Bills may be an attempt to resolve the problem, but they may also be signals politicians send to voters or donors. Further, whether any given bill becomes law may depend on which party controls Congress [14]. That said, the fact that we find an effect on concentration not just on bills proposed, but also passed, suggests that the impact is broader than just interest alone.

One could wonder whether there is bias in the measurement and/or recording of the mass shootings. If mass shootings that occur in quick succession raise attention to the issue of gun legislation, for example, maybe such shootings are more likely to receive attention in the press, and thus increase the chance they appear in the dataset. While this is possible, it seems unlikely. First, while not every shooting receives news coverage, mass shootings are not just any shooting. The fact that at least four people are killed makes it much more likely that each incidence is reported. Second, rather than just manually search for examples in the news, we relied on some existing datasets built solely for the purpose of tracking mass shootings (e.g., from Mother Jones). These data sources have been updated and expanded multiple times, and cross referencing them with other sources decreases the chance that any examples have been omitted.

One could also wonder whether the effect of concentration is truly causal. Maybe concentration of mass shootings is driven by other issues such as poverty and inequality, for example, which also directly affect gun legislation. This seems unlikely, however, for a few reasons. First, mass shootings are not the same as gun violence more generally. While poverty and inequality may certainly increase crime, the use of guns, and thus gun violence, by definition, mass shootings involve at least four firearm fatalities, excluding the offender(s), and do not take place in the process of some unlawful act such as burglary or organized crime. Consequently, poverty and inequality certainly impact crime and gun use in general, individual level factors like mental health history may matter more for mass shootings [30]. Indeed, prior work has argued that mass shootings are “plausibly random occurrences [14]. Second, and along those lines, while factors like poverty and inequality certainly shift over time, in the short term they are usually quite stable. Concentration, on the other hand, is quite volatile over the observation period. Consequently, while poverty or inequality might shape the overall number of mass shootings, it is less clear that they would impact the exact concentration. If four mass shootings happen in a year, it’s unclear why poverty or inequality would make them more likely to be concentrated in time. Indeed, the exact concentration seems more likely to be exogenous.

### Ancillary analysis

To further test whether potential causal nature of the relationship between concentration and bills, we performed path analysis. Path analysis is a form of structural model that quantifies directed dependencies in a system of variables. Specifically, we focus on the billing equation in (3) and test the causal effect of concentration on the number of bills (i.e. endogenous variable) while also including the attention construct, time since last bill, shooter age, number of shooters as exogenous variables i. There was a significant effect of concentration on the number of bills (b = .004, z = 2.97, p = .003) supporting the notion that concentration is causing additional bills.

That said, to rule out alternatives, directly demonstrate causality, and test generalizability, we conduct an experiment.

### Follow up experiment

Empirical analysis of hundreds of mass shootings is consistent with the notion that concentration encourages action. That said, to provide even more direct evidence of concentration’s causal impact, we conducted an experiment. We manipulated the temporal concentration of information related to a social issue, and test whether that influences support. Specifically, consumers are often exposed to information about different social problems through the news, so we gave participants headlines about different social issues. We manipulated the concentration of articles around a specific issue (i.e., sexual assault) and test whether that impacts willingness to sign a petition or donate to support the cause.

Note that one could wonder whether the effects observed so far are somehow restricted to mass shootings. Maybe there is something unique about shootings that leads concentration to encourage action, but that the effect would not hold for other social issues. While this seems unlikely, to test the generalizability of the effect, we consider a different social issue (i.e., sexual assault).

### Method

Five hundred and fifty-eight (270 women; M_age_ = 36.56) participants completed a study on Amazon Mechanical Turk for $1 (procedure was approved by University of Pennsylvania IRB and participants provided written consent). Participants read that the study was about “what news articles people are reading, and which news articles people think are important,” and we randomly assigned participants to one of two concentration conditions (control vs. concentration) in a between subjects design. All participants viewed the same 40 news headlines covering eight different social justice issues (ageism, sexual assault, gun control, free speech, racism, education, healthcare, gay rights), which were obtained from real news stories.

We manipulated concentration through the order in which the headlines were presented. In the control condition, the five sexual assault headlines were randomly interspersed throughout the set of 40 headlines. In the concentration condition, the five sexual assault headlines were concentrated, appearing one after the other.

After reading all the headlines, participants completed the key dependent variable. Participants were asked whether they would “be willing to sign a petition related to any of these social issues in a future study” and were invited to “check all that apply” with an option for each of the eight social justice issues covered in our study’s headlines. We analyzed whether participants checked “sexual assault.”

### Results and discussion

As predicted, concentration encouraged action. A logistic regression found that concentration increased the number of participants who were willing to sign the petition in protest of sexual assault (*M*s = 61% vs. 51%), *b* = .42, *SE* = .18, *OR* = 1.52, *t* = 2.31, *p* = .02, 95% CIs [.07, .77].

Results of the experiment underscore the findings observed in the field: Concentration encourages action. Concentrating information about an important social issue (i.e., sexual assault), made participants more likely to sign a petition to support that cause. This holds even given the volume of information was the same across conditions, and thus only concentration was varied. Experimentally manipulating concentration underscores it causal impact. Further, the fact that the effect held in another important social justice domain speaks to its generalizability.

## General discussion

Many pressing social problems deserve attention, yet few receive the attention they deserve. A multi-method investigation demonstrates that *the relative timing* of related events plays an important role in shaping action. Analysis of 40 years of mass shootings and gun control legislation suggests higher concentration of shooting incidences encourages response. Even after controlling for critical shooting incidence characteristics such as frequency and number of fatalities, gun control legislation is more likely to occur when mass shootings are concentrated in time, rather than spread out. A standard deviation increase in the concentration of shootings within a month leads to a 25.4% increase in the number of bills proposed. This effect holds controlling for a variety of other factors, as well as at different aggregation levels of the data.

Further, a follow up experiment underscores concentration’s causal impact. Directly manipulating concentration demonstrates that it encourages action. Concentrating attention to sexual assault increased people’s willingness to sign a petition and donate to the cause.

While we focused on gun control and sexual assault, similar dynamics may have encouraged the rise of other social movements such as #MeToo and historical legislation such as the 1964 Civil Rights Act [19]. Take brand boycotts, for example. While product recalls, unfair labor practices, or other negative events may increase the chance of consumer backlash, our results suggest that such backlash should be particularly likely when such negative events are concentrated in time. While multiple missteps should increase the likelihood of negative sentiment or broader boycotts, these responses should be more likely when such missteps occur in short succession.

Various psychological mechanisms may contribute to these effects. Concentration may make events more accessible, which could increase perceived importance [31]. Events tend to decay in memory [32], but concentration may counteract this decay, making action more likely. Rapid changes can direct attention [33] and rapidly occurring events may similarly encourage attention to social problems. The negative affect from aversive events may also decay over time, but concentration may encourage more enduring negative affect, which may increase action [34]. Concentration may also increase beliefs about the underlying likelihood of events moving forward, which may also increase action.

Our findings have important practical and policy implications. While past work has focused on the importance of message framing in driving action, we provide evidence for the importance of the concentration of related events in the attention social problems receive. Consequently, those looking to draw attention to particular problems and encourage action, may want to harness concentration. Social justice campaigns, for example, may be more successful if they communicate problematic events in high-frequency concentrated bursts rather than in steady and periodic messages. Or media and news channels could highlight the concentration of incidences that relate to a social issue in their attempt to generate awareness and build attention around it. More broadly, policymakers and organizers may want to attend to the role of timing in cultural change, and the potential for infrequent and low-concentration events to be neglected. Recognizing the role of concentration may help more movements inspire action.

Future research might examine moderators of this effect across different domains. Instances of sexual harassment might be more likely to inspire change when spread across industries, for example, because it suggests the problem is more widespread. Subsequent work might also test other outcomes. Legislation may be a conservative test (given the threshold for action), but cultural change can also take the form of grassroots action or social media attention. Things like media coverage may also mediate this effect. Concentrated events may encourage media attention, which in turn encourage legislative and grassroots action.

More broadly, similar effects may drive other social phenomenon, like why things catch on. Some products, services, and ideas catch on while others fail. While features of the thing itself obviously matter, patterns of social influence may also play a role. While hearing about something from others likely encourages adoption, and hearing from more others encourages it even further, concentration may also contribute. Hearing from multiple others in a shorter time period should be more likely to encourage adoption and action.

## Supporting information

S1 Table Descriptive statistics. (DOCX)

S2 Table Model validation. (DOCX)

S3 Table Robustness to aggregation level. (DOCX)
