# Supplementary material for: Catalyzing social change: Does concentration encourage action?
Source: PLoS One. 2022 Dec 28;17(12):e0277934. doi: 10.1371/journal.pone.0277934 (PMC9797062; doi:10.1371/journal.pone.0277934)
Supplement: S1 Table — (DOCX) [file pone.0277934.s001.docx]

**Table S1:** Descriptive Statistics

|  | # Bills | # Shootings | # Shooters | Avg Shooter Age | # Fatalities | Avg days b/w shootings | Months since Last Bill |
| --- | --- | --- | --- | --- | --- | --- | --- |
| Mean | 1.49 | 0.47 | 1.50 | 35.45 | 9.57 | 81.50 | 0.92 |
| SD | 2.53 | 0.80 | 0.79 | 10.99 | 9.15 | 91.93 | 1.78 |
| Kurtosis | 19.38 | 3.98 | 2.46 | -0.20 | 16.38 | 5.75 | 11.37 |
| Skewness | 3.63 | 1.92 | 1.61 | 0.31 | 3.65 | 2.23 | 2.94 |
| Min | 0 | 0 | 1 | 15 | 2 | 4 | 0 |
| Max | 21 | 5 | 5 | 66 | 65 | 509 | 12 |
| Sum | 713 | 230 | 239 | NA | 1521 | NA | NA |
| Count | 480 | 480 | 159 | 159 | 159 | 159 | 249 |
