# Supplementary material for: Catalyzing social change: Does concentration encourage action?
Source: PLoS One. 2022 Dec 28;17(12):e0277934. doi: 10.1371/journal.pone.0277934 (PMC9797062; doi:10.1371/journal.pone.0277934)
Supplement: S2 Table — (DOCX) [file pone.0277934.s002.docx]

**Table S2:** Model Validation

|  | Proposed Model |  |  |  |  |  |  |  |  |
| --- | --- | --- | --- | --- | --- | --- | --- | --- | --- |
| Shootings | Concave | | | Linear | | | Convex | | |
| Fatalities | Convex | Linear | Concave | Convex | Linear | Concave | Convex | Linear | Concave |
| LL | **-614.18** | -631.98 | -625.49 | -625.75 | -627.09 | -626.09 | -629.29 | -628.19 | -639.8 |
| AIC | **1246.36** | 1281.96 | 1268.98 | 1269.5 | 1272.18 | 1270.18 | 1276.58 | 1274.38 | 1297.6 |
| BIC | **1283.60** | 1319.75 | 1306.77 | 1307.29 | 1309.97 | 1307.97 | 1314.37 | 1312.17 | 1335.4 |
