# Supplementary material for: Catalyzing social change: Does concentration encourage action?
Source: PLoS One. 2022 Dec 28;17(12):e0277934. doi: 10.1371/journal.pone.0277934 (PMC9797062; doi:10.1371/journal.pone.0277934)
Supplement: S3 Table — (DOCX) [file pone.0277934.s003.docx]

**Table S3:** Robustness to Aggregation Level

|  | **Triweekly** | **Bimonthly** |
| --- | --- | --- |
| **Concentration** | **.002*** | **.004*** |
| Number of Shootings | .32** | .09 |
| Carryover Effect | .76*** | .97*** |
| Number of Fatalities | .94*** | .23* |
| Time Since Last Bill | .01*** | .00 |
| Average Shooter Age | -.02** | -.01* |
| Number of Shooters | .75*** | .87* |

*p < .05, **p < .01, p < .001
